# Dual Role of CXCL10 as Conductor of Cellular Trafficking during Type 1 Diabetes

**Published:** 2007-06

**Authors:** Urs Christen, Edith Hintermann

**Affiliations:** *Pharmazentrum Frankfurt / Zentrum für Arzneimittelforschung, Entwicklung und Sicherheit (ZAFES), Johann Wolfgang Goethe University, Frankfurt am Main, Germany*

**Keywords:** IP-10, TNFα, LCMV, chemokine, cytokine

## Abstract

One possible way of how autoimmune disease can be initiated is by infection with a foreign pathogen. Especially viruses are thought to act as triggering factors, inducing a detrimental attack against ‘self’ by the immune system of a susceptible host because of two major reasons. First, viruses cause a massive inflammation of the infected tissue and therefore initiate the infiltration of a broad variety of leukocytes, including potentially ‘self’-reactive lymphocytes. Second, some viruses have been demonstrated to bear molecules with a strong structural similarity to host components. The existence of such a ‘molecular mimicry’ may elicit an immune response, which is initially generated in defense against the invading pathogen but in a second wave targets similar structures of the host. In the present review we will reflect on the dual role of inflammatory factors during this process and discuss possibilities of how such a detrimental second wave of the immune system can be blocked. Most of the presented data have been obtained from animal models that integrate the concept of molecular mimicry. They use transgenic mice expressing a defined model protein specifically in a target tissue and a virus containing either an identical or a similar protein as a triggering factor.

## INTRODUCTION

Virus infections elicit an innate anti-viral defense mechanism in the host that includes the upregulation of a broad variety of inflammatory factors, such as prostaglandins, leukotriens, chemokines and cytokines. Chemokines are considered as conductors of cellular trafficking, due to their ability to chemically attract different subpopulations of leukocytes to the site of inflammation (1). At least 50 different chemokines have been characterized to date and many bind to more than one cellular receptor. In addition, many receptors can interact with different chemokine ligands. Thus, chemokines together with other cytokines and their respective receptors form a complicated network, which is partially overlapping in its function and in part self-propelling in its activation (1). Therefore, it is difficult to identify single factors in this network that may play a crucial role in the development of autoimmune diseases, such as type 1 diabetes (T1D). Nevertheless, neutralization of some of those inflammatory factors has been successfully employed for therapy of some autoimmune diseases. For example, blockade of tumor necrosis factor-α (TNFα) is now widely used to treat human rheumatoid arthritis (2). However, several lines of evidence indicate a dual role for chemokines and cytokines in the etiology of autoimmune diseases depending on the time and location of their expression. Here, we will review some of our studies that focused on dissecting the precise mechanism of how TNFα, interferon-inducible protein of 10kDa (IP-10, CXCL10) or general virus infections can induce, accelerate or abrogate autoimmune disease in an animal model for T1D.

### The RIP-LCMV mouse model for T1D: Virus induced breakdown of self-tolerance

More than 200 million people worldwide suffer from diabetes. It is estimated that 5-10% thereof have type 1 diabetes (T1D), which is characterized by the autoimmune destruction of the insulin-producing β-cells of the islets of Langerhans in the pancreas. Even though the lack of endogenous insulin can be replaced by injections of exogenous insulin, T1D is still a tremendous health problem. As a consequence of the imbalanced blood glucose homeostasis, patients with T1D often develop severe complications, such as cardiovascular diseases, retinopathy (blindness), neuropathy and nephropathy. Based on an extensive body of literature, which associates virus infections with autoimmune diseases [for review see (3-5)], the following scenario for the pathogenesis of T1D was hypothesized and formed the basis for establishing a virus-induced animal model for T1D to study the etiology and pathogenesis of human diabetes. First, restricted but low level expression of self, environmental, or viral antigen occurs in β-cells of the islets of Langerhans. This event by itself does not cause disease, since the host is hypo-responsive or tolerant to the antigen. Tolerance can be achieved by thymic expression of the self or viral antigen or through peripheral tolerance mechanisms (6-10). Later in life, a triggering event occurs, which is exposure to the same environmental factor, infection with the same virus or pathogen or with cross-reacting antigenic determinants. The result is an immune response to the pathogen that eventually localizes to the β-cells and progresses to T1D after a lag period. This scenario was experimentally reconstructed in the late 1980s by the laboratories of Michael Oldstone (11) and Rolf Zinkernagel (12). Both groups used a rat insulin promoter (RIP) to create separate lines of transgenic mice whose pancreatic β-cells expressed either the nucleoprotein (NP) or the glycoprotein (GP) of the lymphocyte choriomeningitis virus (LCMV) as defined target antigen. The expression of the target (self)-antigen does not lead to β-cell dysfunction, islet cell infiltration, hyperglycemia, or spontaneous activation of autoreactive (anti-LCMV) lymphocytes (13). However, infection with LCMV results in autoimmune diabetes in >95% of RIP-LCMV mice. In contrast, non-transgenic littermates never develop diabetes or insulitis after LCMV challenge (13). Hence, the RIP-LCMV model has become a very useful tool to study the etiology and the mechanisms of human autoimmune diabetes and to evaluate possible treatments, such as blockade of specific inflammatory factors (14, 15), oral tolerance induction (16) or DNA-vaccination (17). Besides having a clearly defined initiation point (LCMV-infection), the advantage of the RIP-LCMV system over other established models for T1D, such as the NOD mouse, is the presence of extensively characterized target antigens (GP, NP). The immune response against these target antigens can be visualized using flow cytometry by stimulation of splenocytes with LCMV-peptides or direct staining of CD8 T-cells with MHC class I-peptide tetramers (18). In addition, we recently demonstrated tracking of LCMV-specific CD8 T-cells by *in situ* MHC class I-peptide staining of quick-frozen tissue sections (19).

### Immunopathogenesis of β-cells destruction in the RIP-LCMV model

**Figure 1:**
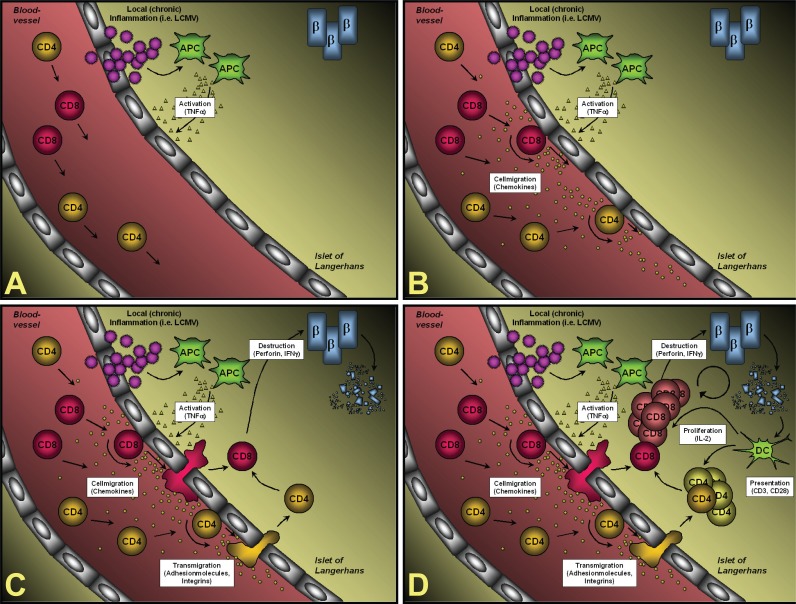
Immunopathogenesis of β-cell destruction in the RIP-LCMV model.

Figure 1 summarizes the individual steps on the way from the initial virus infection of the pancreas to the ultimate destruction of most of the β-cells, which is the cause for overt diabetes. (A) Infection of RIP-LCMV mice causes a local inflammation in the pancreas. Note that LCMV in fact infects other organs, such as the liver or the kidney, as well; here we will however focus on the target organ that expresses the viral transgene, the pancreas. Initially it is very likely that resident macrophages are activated and predominantly release TNFα into the local microenvironment. Blockade of TNFα immediately after LCMV-infection prevents the development of T1D (14). (B) TNFα signaling results in the activation of the local endothelium, which in turn releases chemokines. In particular CXCL10 (IFNγ-inducible protein of 10 kDa, IP-10) is generated at high levels very early after infection (15). Alternatively, CXCL10 might be expressed by β-cells themselves (20). CXCL10 is captured by cell surface heparan sulfate proteoglycans from the fluid phase and forms a concentration gradient around the site of infection (21). Interaction of the captured CXCL10 with its cellular receptor, CXCR3, on rolling lymphocytes induces the surface expression of integrins, such as ICAM-1 (22). Blocking of CXCL10 with neutralizing antibodies at this stage abrogates T1D (see below) (15). (C) Upon integrin-mediated firm attachment, the lymphocytes transmigrate through the endothelium into the pancreas and the islets of Langerhans. CXCL10 has been reported to attract monocytes, T-cells and NK cells (23, 24). However, CXCR3, the only cellular receptor for CXCL10 identified to date, is predominantly found on activated T-cells of the more aggressive Th1-type (25, 26). In addition, we found that after LCMV infection more than 95% of LCMV-specific CD8 T-cells express CXCR3 (15). Within the islets of Langerhans the autoaggressive T-cells start destroying some of the β-cells in a perforin- and IFNγ-dependent manner (27, 28). At that time the mice are not fully diabetic, since the remaining β-cells are still able to compensate for the destruction of some β-cells by producing more insulin per individual β-cell. (D) However, the perforin-mediated β-cell lysis is thought to produce β-cell debris, which is then processed and presented by antigen-presenting cells (APCs), such as infiltrating dendritic cells (DCs). Such an enhanced presentation of LCMV-epitopes and other β-cells-antigens causes further activation and proliferation of β-cell-specific T-cells, which subsequently execute the destruction of the majority of β-cells resulting in overt diabetes. At this stage the production of IFNγ is critical and T1D does not occur in IFNγ-deficient mice (29). The duration of the whole destructive process is dependent on the number and affinity of LCMV-specific precursor T-cells: Transgenic lines expressing the LCMV-GP transgene exclusively in the β-cells manifest rapid-onset diabetes, usually 10-14 days after viral challenge (13). T-cells develop normally and have equivalent cytotoxic T lymphocyte (CTL) activity to splenic lymphocytes from non-transgenic age- and sex-matched littermates. In these lines the high systemic numbers of auto-aggressive CD8 T-cells are sufficient to induce diabetes and do not require help from CD4 T-cells. In contrast, in lines expressing the LCMV-NP transgene in both the β-cells and the thymus, T1D takes longer to occur after LCMV challenge. Several lines of evidence indicate that anti-self (viral) CTL were of lower affinity and that CD4 T-cells were essential to generate anti-self (viral) CD8 T-cell-mediated T1D of RIP-LCMV-NP transgenic mice (13).

### Cytokines: Dual role for TNFα depending on the time of expression

As mentioned above, blocking of TNFα with a TNFR55-IgG1 fusion protein (30) immediately after LCMV-infection abrogates T1D (14). However, the peak of TNFα expression in the pancreas is around day 7 post-infection (14, 15). Neutralization of TNFα around day 7 or later failed to prevent T1D. Therefore, we were asking what the role of TNFα is at a later stage of immunopathogenesis. To answer this question, we decided to overexpress TNFα specifically in the β-cells using the tTA-system (31), which allows to precisely manipulate islet-specific TNFα expression. Briefly, by crossing RIP-LCMV-GP mice with Tet-TNFα mice (32) we were able to control (i) the onset of the autoimmune process by infection with LCMV and (ii) the expression of TNFα in the β-cells by removal of doxycycline from the diet. Tet-TNFα mice express TNFα via the tTA-system under the control of a tetracycline sensitive promoter system (31). The resulting RIP-GP-TNFα mice (14) were therefore bred in presence of the tetracycline derivative doxycycline (Dox) to block transgenic TNFα expression. Dox was removed at several times after the onset of the autoimmune process (infection with LCMV) to induce β-cell specific TNFα expression.

As expected, the exact time of TNFα expression was very important for the influence of TNFα on diabetes pathogenesis in LCMV-infected RIP-GP-TNFα mice and revealed a dual role of TNFα. Early expression (at the time of LCMV infection) slightly enhanced the frequency of diabetic mice (14). In contrast, late expression (at day 10-14 post-infection - a time when most animals are already diabetic) resulted in a significant decrease in T1D incidence. At the same time the frequency of ‘revertant’ mice was increased. Such ‘revertant’ mice were initially diabetic (blood glucose >300 mg/dl) at weeks 2 and 3 post-infection, but reverted persistently to non-diabetic blood glucose values (<200 mg/dl) after week 4-5 (14). The occurrence of ‘revertant’ mice was an exciting phenomenon since TNFα could not only reduce the frequency of autoimmune disease but also abrogate the actual ongoing autoimmune process at a time when its clinical features were already obvious. Mechanistically, we found that late TNFα-expression results in an hyperactivation and subsequent apoptosis of autoaggressive, LCMV-specific CD8 T-cells in ‘revertant’ RIP-GP-TNFα mice (14). Late expression of TNFα in the RIP-GP-TNFα mouse model reduced the incidence of diabetes to ~35% and, additionally, reversion from clinically overt diabetes to normoglycemia occurred in up to 50% of the mice. An interesting question is why not all mice ‘reverted’ to normal blood sugar levels. One scenario might be that a combination of early diabetes onset and late TNFα availability would lead to enhanced physiological stress to β-cells, preventing them from producing transgenic TNFα and therefore reducing the possibility of TNFα induced apoptosis of autoreactive T cells.

### Chemokines: Dual role for CXCL10 as conductor of the autoaggressive immune response towards and away from the pancreas

Cellular trafficking to the site of infection is predominantly orchestrated by chemokines that attract and activate cells of the host’s immune system with the goal to eliminate the foreign pathogen from the organism. Among chemokines, CXCL10 stands out of the mass, because of its massive and unique expression very early after infection of mice with LCMV. CXCL10 mRNA was detected as early as day 1 after infection and was elevated by more than 400-fold (15). In contrast, expression of the other two ligands of CXCR3, CXCL9 (Mig) and CXCL11 (I-TAC), followed a delayed kinetics. Expression of CXCL9 mRNA was maximally upregulated (~30-fold) between days 2 to 7 after LCMV infection and CXCL11 mRNA was only upregulated 3-fold over levels expressed in uninfected controls. Importantly, the levels and kinetics of expression of all CXCR3 chemokine ligands induced by acute LCMV infection was equivalent in both C57BL/6 wildtype and RIP-LCMV-GP transgenic mice indicating that CXCR3 chemokine ligands are expressed as a direct result of the infection of the pancreas by LCMV. Significant expression of CXCR3 was detected as early as day 7 after LCMV infection (15). Since CXCR3 is expressed predominantly on activated T-cells, it is most likely that CXCR3 expression in the pancreas is due to the infiltration of activated T-cells that occurs around days 7 to 10 after LCMV infection (33). Other chemokines like CCL5 (RANTES) showed a kinetic profile different than the CXCR3 chemokines and only modest elevations of CCL2, CCL11, and CXCL1 were noted, with no detectable expression of CCL1, CCL4, and CXCL2 (15). Thus, because of its unique appearance early after infection of the pancreas, CXCL10 might be a prime target for a therapeutic neutralization. Indeed, blocking of CXCL10 with neutralizing antibodies at the peak of its expression abrogated T1D in the RIP-LCMV mouse (15). Mechanistically, CXCL10 neutralization resulted in the blockade of trafficking of autoaggressive T-cells to the pancreas and therefore prevented the destruction of the insulin-producing β-cells.

It is important to note that CXCL10 neutralization had to occur precisely at the time of its peak expression. Administration of neutralizing anti-CXCL10 antibodies at an earlier (i.e. before infection) or later (i.e. 7 days post-infection) time failed to interfere with the autodestructive process. Similarly, blockade of other chemokines, such as CCL5 had no influence on the outcome of T1D (15). Hence, neutralization of one chemokine (CCL5) out of many other inflammatory factors that have been expressed in parallel was not successful in preventing T1D, possibly due to a partial redundancy within the cytokine / chemokine network. In contrast, elimination of the unique critical factor (CXCL10) at the very beginning of the starting immunopathogenesis was a successful therapy to prevent the imprinting of a pattern for the subsequent autoaggressive immune response.

The key role of CXCL10 could be further confirmed in RIP-CXCL10 transgenic mice that overexpress CXCL10 specifically in the islets of Langerhans (34). RIP-CXCL10 mice have spontaneous peri- and intra-islet infiltration by various lymphocytes but do not develop spontaneous T1D (34). However, when crossed to the RIP-LCMV transgenic lines, RIP-LCMV-NP x RIP-CXCL10 double transgenic mice displayed a massively accelerated diabetes onset after LCMV infection (34). In contrast, no change in diabetes onset was observed in RIP-LCMV-GP x RIP-CXCL10 double transgenic mice possibly due to the inherent nature of the RIP-LCMV-GP line, which is already developing diabetes with a fast-onset kinetics (13, 34). The observed acceleration was predominantly caused by the presence of much higher numbers of autoaggressive CD8 T-cells in the islets of Langerhans at an early time after LCMV-infection. Obviously, the presence of cellular infiltrates before LCMV-infection (spontaneous insulitis) kick started the autodestructive process by providing both inflammatory mediators (chemokines, cytokines) as well as β-cell antigen-specific cytolytic T-cells.

### Dual role of virus infections on the immunopathogenesis of T1D

As described above, CXCL10 plays a key role in orchestrating the cellular trafficking towards the site of infection and subsequently imprints a pattern for the detrimental autoaggressive immune response against the islet of Langerhans. However, we could also demonstrate that expression of CXCL10 outside of the pancreas can actually oppose an ongoing autoaggressive destruction of β-cells. It is known since the late 1980s that infection by LCMV blocks T1D in the NOD mouse (35). We wanted to analyze the mechanisms behind this protection by virus infection in more detail. Therefore, we used LCMV-infected RIP-LCMV-NP mice that are normally prone to develop T1D within 2-6 month post-infection and infected these mice for a second time with different LCMV strains at week 4 after the primary infection. Infection with one particular virus strain, LCMV-Pasteur, indeed prevented T1D in the RIP-LCMV mice (36). LCMV-Pasteur was different from other viruses tested, in that it grew to much higher titers in the pancreatic draining lymph node (PDLN) compared to the pancreas. In parallel, a massive expression of inflammatory factors, especially of CXCL10 was observed in the PDLN (36). Importantly, we also found a significantly reduced degree of cellular infiltrates in mice that were re-infected with LCMV-Pasteur compared to mice that did not receive a secondary infection. At the same time (day 3 post-secondary infection), a much higher number of autoaggressive T-cells were dying of apoptosis in the PDLN (36). These data indicate that autoaggressive T-cells migrated out of the pancreas towards the highly inflamed PDLN, where they died by hyper-activation-induced apoptosis. In this scenario the PDLN acted just like a filter that removes autoaggressive T-cells from the circulation (37).

It is important to note here that not all viruses have a protective property. The role of viruses in autoimmune diseases is controversially discussed and although many human autoimmune diseases have been associated with a broad variety of different viruses, it is often difficult to find direct prove for certain viruses to induce or prevent autoimmune disease (37). One reason for this lack of direct evidence is that patients suffering from autoimmune diseases and healthy individuals alike undergo multiple infections during their lifetime. Most viruses have been cleared by the time of disease diagnosis. Thus, virus infections can be considered as ‘hit and run’ events that leave no precise footprints to document the patient’s history of prior infections. Second, genetic factors are not only directly responsible for disease susceptibility but also influence the anti-viral immune response itself. Third, certain infections protect from autoimmunity rather than enhance it (see above). Forth, infections might not directly initiate autoimmunity, but will rather accelerate a preexisting autoimmune condition to progress to clinical disease. Thus, multiple sequential events may be necessary to precipitate disease. In such a scenario molecular mimicry might be an important factor that has to be considered. For example, when we re-infected RIP-LCMV-NP mice with a heterologous virus (Pichinde Virus, PV) at week 4 after LCMV-infection, we observed a massive acceleration of T1D with similar kinetics as observed in RIP-LCMV-NP x RIP-CXCL10 mice (38). The reason for this exacerbated course of disease lays in the existence of a structural similarity between two subdominant epitopes of LCMV and PV (39). Such a natural molecular mimicry caused a massive expansion of a T-cell population, which is normally (after single infection with either LCMV or PV) negligible, playing only a minor role in anti-viral defense as well as in the autoimmunity. Thus, the total number of autoaggressive, islet-antigen-specific lymphocytes, was significantly enhanced after heterologous infection with LCMV and PV (38, 39) and T1D was accelerated. This example illustrates the complexity involved in the etiology of autoimmune disease and suggests that the entire infectious history of each human individual might determine the overall immune status that results in autoimmune disease or not.

## SUMMARY

**Figure 2:**
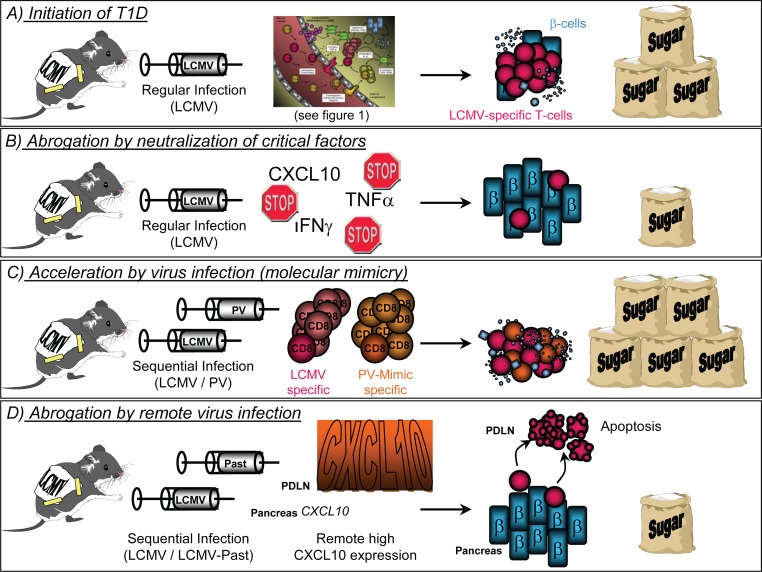
Dual role of chemokines, cytokines and virus infections in T1D.

It is generally believed that autoimmune diseases occur as a detrimental combination of genetic susceptibility and environmental factors. Viruses are considered to be the prime candidates to act as such triggering factors, because of their ability to induce a strong inflammatory response and since they drive the anti-viral immune response towards a more aggressive, cytolytic domination. Key inflammatory mediators, such as TNFα or CXCL10, play a dual role in the development of autoimmune disease, depending on the time and location of their expression. First: TNFα is necessary to initiate the inflammatory process and blocking of TNFα inhibits the development of disease (Figure 2A and B). In contrast, when expressed late, at a time when the autodestructive process is already ongoing, TNFα expression in the islets of Langerhans is protective by inducing apoptosis of autoaggressive T-cells (14). Second: The unique expression of CXCL10 after LCMV infection of the pancreas imprints on one hand a pattern for the subsequent development of autoimmune disease and neutralization of this one single key factor precludes disease (Figure 2A and B). On the other hand, the same ‘danger signal’ abrogates T1D when expressed at an auxiliary site causing autoaggressive lymphocytes to leave the target organ (Figure 2D). However, in both cases CXCL10 turned out to be the conductor of the cellular trafficking into or out of the pancreas (15, 34, 36). Lastly, viruses by themselves play a dual role in the etiology of autoimmune diseases: By molecular mimicry of ‘self’-components of the host or of structures on heterologous pathogens that have infected the host at an earlier time, viruses can initiate or accelerate the autoimmune process (Figure 2C). In contrast, viruses can impede the progress of disease when infecting an auxiliary site by filtering autoaggressive T-cells out of the circulation and the immune repertoire of the host (Figure 2D). Considering these dual roles of the key components mediating the process of autoimmunity, the importance of a well-calculated therapy becomes apparent. However, in order to design such a therapeutic regimen it is vital to know the precise mechanistic details behind the auto-destructive processes. Only with this knowledge a therapeutic neutralization of those key factors that drive the pathogenesis of human autoimmune diseases can be applied successfully.
